# Effect of Resveratrol Supplementation on Intestinal Oxidative Stress, Immunity and Gut Microbiota in Weaned Piglets Challenged with Deoxynivalenol

**DOI:** 10.3390/antiox11091775

**Published:** 2022-09-08

**Authors:** Yueqin Qiu, Xinzhi Nie, Jun Yang, Li Wang, Cui Zhu, Xuefen Yang, Zongyong Jiang

**Affiliations:** 1 Institute of Animal Science, Guangdong Academy of Agricultural Sciences, Guangzhou 510640, China; 2 State Key Laboratory of Livestock and Poultry Breeding, Guangzhou 510640, China; 3 Key Laboratory of Animal Nutrition and Feed Science in South China, Ministry of Agriculture and Rural Affairs, Guangzhou 510640, China; 4 Guangdong Provincial Key Laboratory of Animal Breeding and Nutrition, Guangzhou 510640, China; 5 Maoming Branch, Guangdong Laboratory for Lingnan Modern Agriculture, Guangzhou 510640, China; 6 School of Life Science and Engineering, Foshan University, Foshan 528225, China

**Keywords:** deoxynivalenol, intestinal inflammation, gut microbiota, *Lactobacillus* *gasseri*, oxidative stress, piglets, resveratrol

## Abstract

(1) Background: Deoxynivalenol (DON) is a general mycotoxin that induces severe intestinal barrier injury in humans and animals. Resveratrol (RES) efficiently exerts anti-inflammatory and antioxidant effects. However, the information regarding RES protecting against DON-induced oxidative stress and intestinal inflammation in piglets is limited. (2) Methods: A total of 64 weaned piglets (Duroc × (Landrace × Yorkshire), 21-d-old, barrow) were randomly allocated to four groups (eight replicate pens per group, each pen containing two piglets) for 28 d. The piglets were fed a control diet (CON) or the CON diet supplemented with 300 mg RES/kg diet (RES group), 3.8 mg DON/kg diet (DON) or both (DON+RES) in a 2 × 2 factorial design. (3) Compared with unsupplemented DON-challenged piglets, RES supplementation in DON-challenged piglets increased ileal villus height and the abundance of ileal *SOD1*, *GCLC* and *PG1*-5 transcripts and *Muc2* protein (*p* < 0.05), while decreasing the mRNA and proteins expression of ileal *IL-1β*, *IL-6* and *TNF-α*, and malondialdehyde (MDA) levels in plasma and ileum in DON-challenged piglets (*p* < 0.05). Moreover, the abundances of class *Bacilli*, order *Lactobacillales*, family *Lactobacillaceae* and species *Lactobacillus* *gasseri* were increased in DON-challenged piglets fed a RES-supplemented diet compared with those in DON-challenged piglets(*p* ≤ 0.05). (4) Conclusions: our results indicated that RES supplementation in DON-challenged piglets efficiently attenuated intestinal inflammation and oxidative stress and improved gut microbiota, thereby alleviating DON-induced intestinal barrier injury.

## 1. Introduction

Deoxynivalenol (DON) is a major mycotoxin detected in a wide range of food crops and livestock feed [1], causing toxicity to human health and animal productivity [2]. Accumulating evidence has reported that DON infection induces a severe dysfunction of intestinal barrier function [3,4]. Previous studies demonstrated that a DON-contaminated diet induces intestinal anti-inflammatory cytokines, decreases the gene and protein expression related to antioxidants and disrupts intestinal tight junction in piglets [1,5,6,7,8,9,10]; however, the effect of DON on the expression of intestinal antimicrobial peptides and mucin protein that provides defenses against toxins invasion into intestinal epithelium needs further exploration.

At present, preventing DON-induced intestinal oxidative damage and inflammation may be a potential strategy to protect against intestinal injury. Previous studies showed that polyphenols extracted from plants could alleviate intestinal oxidative stress damage and inflammation, prevent invasive and pathogenic microorganisms, enhance epithelial integrity and improve the growth performance of weanling piglets [5,11,12,13,14,15,16]. Resveratrol (RES), an important member of polyphenols, which is primarily extracted from grapes, red wine and berries [17], attracted tremendous attention in swine production due to its protective effects on anti-inflammatory and antioxidant, and growth performance improvement of weaned piglets [5,11,13,18]. A previous study found that RES and its derived metabolites could be detected with a large amount in the gastrointestinal tract 6 h after RES administration [19], which could explain the mechanisms underlying the beneficial effects of RES on the intestinal barrier functions in experimental animal models [11,18,20]. In vitro studies showed that RES protects against oxidative damage and intestinal inflammation through nuclear factor E2-related factor 2 (NRF2) activation and nuclear factor kappa B (NF-κB) inhibition [10,21,22]. However, only a few studies confirmed that dietary RES supplementation in DON-challenged piglets suppressed intestinal inflammatory response and oxidative stress [5].

The gut microbiota plays an important role in maintaining intestinal barrier homeostasis. Many studies have demonstrated that DON challenge has a deleterious effect on the intestine; however, only a few studies determined the effect of DON on the gut microbiota of piglets [23,24,25,26]. Previous studies reported that polyphenols modulate the colon microbiota composition, which contributes to maintaining gut health. Several studies found that polyphenols supplementation effectively changed the composition of gut microbiota, elevating the growth of beneficial bacteria while inhibiting the abundance of pathogenic bacteria in pigs and other animal models [11,15,16,27,28]. Meng et al. reported that supplemental resveratrol increased the relative abundance of butyrate-producing bacteria in weaned piglets [11]. However, the information regarding the effect of RES supplementation in DON-challenged piglets on gut microbiota composition is still limited.

Thus, in the present study, we hypothesized that dietary RES supplementation in DON-challenged piglets alleviated intestinal inflammation and oxidative damage and improved gut microbiota induced by DON challenges. The effects of dietary RES supplementation on intestinal immunity and antioxidant capacity, intestinal morphology and gut microbiota in DON-challenged piglets were investigated.

## 2. Materials and Methods

All animal procedures involved in this study were approved by the Animal Care and Use Committee of the Guangdong Academy of Agricultural Sciences (authorization number GAASIAS-2016-017).

### 2.1. DON-Contaminated Diets Preparation

Fusarium graminearum strain R6576, which is only able to produce DON, was kindly provided by the College of Plant Science and Technology of Huazhong Agricultural University, China. DON-contaminated corn was prepared in accordance with a previous study [29]. Briefly, fusarium graminearum strain R6576 was inoculated on Potato Dextrose Agar (PDA) in an incubator at 28 °C for 7 days. Then aerial mycelia were obtained and cultured in CMC liquid medium. Subsequently, a medium containing conidia was extracted with acetonitrile. Conidia concentration was analyzed using a blood-counting chamber. Then, 25 kg corn was evenly inoculated with 1 L media containing 3 × 10^6^ cfu/mL conidia. The infected corn was then transferred to a plastic bag at room temperature for 7 d. During incubation, moderated ultrapure water was added to provide a moisture content of 80% for infected corn. After incubation, the moldy corns were collected and dried at 65 °C for 24 h, grounded using a hammer mill ground and passed through a 10-mesh screen. The moldy corn contained 7.80 mg DON/kg corn, according to the final determination. To prepare a 3.8 mg DON/kg diet for the DON and DON+RES treatments, moldy corn that replaced 34% normal corn in the basal diet was evenly mixed with the basal diet. In addition, the basal feed and the mycotoxins-contaminated feed (approximately 80 g) were collected and grounded to analyze the main mycotoxins levels in the feed using GC-MS/MS analysis [30]. Briefly, approximately 1 g ground feed was added to a 50 mL polypropylene tube and extracted with 4 mL acetonitrile/water/formic acid (79:20:1, *v*/*v*/*v*) mixture. The tube was shaken using a vertical shaker and then centrifuged at 4000 rpm for 15 min. The supernate was then collected in another tube, and salts (4 g MgSO4 and 1 g NaCl) were added. The tube was immediately closed, vigorously shaken for 1 min and centrifuged for 10 min at 4000 rpm. Then, 2 mL of aliquot was transferred into a 15 mL centrifuge tube, and 100 mg C18 and 600 mg MgSO4 were added. The tube was shaken for 1 min and then centrifuged for 10 min at 4000 rpm, and finally passed through a 0.22 µm filter and transferred into a vial for further GC–MS/MS analysis. The results are shown in Table 1.

### 2.2. Animals and Diets 

A total of 64 weaned piglets (Duroc × (Landrace × Yorkshire), 21-d-old males with an initial body weight of 6.97 ± 0.10 kg) were randomly allocated to 4 groups. Each group consisted of 8 replicate pens, with 2 piglets per pen (n = 16 piglets/treatment). The piglets fed a basal diet were considered the control group (CON), and the other groups were fed the basal diet supplemented with 300 mg RES/kg diet (RES), 3.8 mg DON/kg diet (DON) or 3.8 mg DON +300 mg RES per kg diet (DON+RES) for 28 d. RES (>99.0%) was obtained commercially from Shanxi Ciyuan Biotechnology Co., Ltd. (Xian, China). Based on our previous preliminary experiment, we found that the 300 mg RES/kg diet had a more beneficial effect on the average daily gain of piglets than the 100 and 500 mg RES/kg diet, and the concentration of 300 mg RES/kg diet was therefore chosen in this study. The basal diet was formulated to meet the nutrient recommendations of the National Research Council (NRC) 2012 [31]. Diet compositions and nutrient profiles are shown in Table 2. All pigs had free access to feed and water during the entire feeding period. At the end of the study, the ADG, feed intake, and gain per feed were calculated. (Notes: the experiment in the present study was part of the experiment in Qiu et al., J Anim Sci Biotechnol. 2021; 12: 71, where readers can find the results about growth performance.) One pig from each pen was randomly selected, and the blood was gathered from the anterior vena cava of the selected piglets using heparin lithium anticoagulant tubes. Then, piglets were anesthetized with sodium pentobarbital (40 mg/kg body weight (BW) and sacrificed. The ileum was quickly removed and placed on a cold tray to collect the ileum samples. Two continuous segments were cut from the middle of the whole ileum for histological assay and mucosa collection. Sections of approximately 2.0 cm in length were rinsed with ice-cold PBS and fixed in 4% paraformaldehyde for morphometric evaluation and histochemical staining. Approximately 20 cm section of the ileum was opened longitudinally and cleaned with ice-cold phosphate buffer solution (PBS). Ileal mucosa samples were obtained by scraping with sterile glass microscope slides, snap-frozen in liquid nitrogen and stored at −80 °C for the subsequent analysis.

The plasma samples were collected from the blood by centrifugation at 3500× *g* for 15 min at 4 °C and then stored at −80 °C for further analysis.

### 2.3. Histomorphology Analysis

The fixed samples (ileum) were embedded in paraffin, and 4 μm cross sections from each specimen were mounted on slides coated with polylysine, deparaffinized, rehydrated, and then stained with hematoxylin-eosin (HE) for morphological examination of the ileum. HE-stained slices were scanned using a digital brightfield microscope scanner (Pannoramic 250, 3D HISTECH, Budapest, Hungary). In addition, as for the ileum, fifteen well-oriented and intact villi and adjacent crypts were randomly selected to measure the villus height and crypt depth of each segment using slide viewer software (Case Viewer 2.3, 3D HISTECH, Budapest, Hungary), and the villus height-to-crypt depth ratio (VCR) was calculated.

### 2.4. Enzyme-Linked Immunosorbent Assay (ELISA)

The concentrations of plasma and ileal interleukin 1 beta (*IL-1β*), interleukin 6 (*IL-6*) and tumor necrosis factor-alpha (*TNF-α*), and the contents of ileal *Muc2* and *sIgA* were determined using commercial ELISA kits (Cusabio Biotech Co., Ltd., Wuhan, China) according to the manufacturer’s instructions.

### 2.5. Analyses of Plasma and Intestinal Antioxidant/Oxidant Indices

Approximately 0.1 g of frozen ileal mucosa was homogenized with ice-cold saline (1:9, *w*/*v*) for 2 min. The homogenates were then centrifuged (3500× *g* for 15 min at 4 °C), and the supernatant was collected to analyze the intestinal antioxidant/oxidant index. Total antioxidant capacity (T-AOC), total superoxide dismutase (T-SOD) and the levels of glutathione (GSH) and MDA in the plasma and ileal mucosa were determined using assay kits according to the manufacturer’s protocol (Nanjing Jiancheng Institute of Bioengineering and Technology Nanjing, China).

### 2.6. Quantitative Real-Time PCR (qPCR)

Total RNA was isolated from ileal mucosal samples using a TRIzol reagent (Takara, Tokyo, Japan) in accordance with the manufacturer’s instructions. The purity and concentration of the RNA were identified using a NanoDrop-ND1000 spectrophotometer (Thermo Fisher Scientific Inc., Walldorf, Germany). An amount of 1 μg of total RNA was used to synthesize cDNA using a PrimeScript™II 1st Strand cDNA Synthesis Kit (Takara, Tokyo, Japan). SYBR green I (Takara), 10-fold cDNA dilution and gene-specific primers (Table 3) in a final volume of 20 μL were used to execute qPCR analyses in triplicate. The qPCR were 95 °C × 3 min, followed by 40 cycles of amplification (95 °C × 15 s conditions, 60 °C × 30 s, and 72 °C × 30 s). In the present study, we selected beta-actin (*β-actin*) and GAPDH as the internal control, and the *β-actin* gene was finally used to analyze the data because its abundance was not significantly affected by treatment in the present study (results not shown). The fold changes in target gene expression in piglets fed treatment diets were normalized to *β-actin* and relative to the expression in piglets fed the CON diet; fold changes were calculated for each sample using the 2^−ΔΔCt^ method, where ΔΔCT = (CT_Target_ − CT_*β-actin*_) Treatment − (Average CT_Target_ − Average CT_*β-actin*_) Control.

### 2.7. Gut Microbiome Analysis

As previously reported [32], total DNA from the colonic digesta sample was extracted using the QIAamp PowerFecal DNA Kit (Qiagen, Hilden, Germany) in accordance with the manufacturer’s instructions. The DNA concentration and quality of each sample were evaluated using a Nanodrop 2000 spectrophotometer (Thermo Fisher Scientific, Wilmington, DE, USA). All bacterial 16S rRNA genes covering the V3-V4 region were amplified with the universal forward primer 338F (5’-ACTCCTRCGGGAGGCAGCAG-3’) and the reverse primer 806R (5’-GGACTACCVGGGTATCTAAT-3’). PCR amplicons were purified using the Qiagen Gel Extraction Kit (Qiagen, Duesseldorf, Germany) in accordance with the manufacturer’s instructions. Sequencing libraries were generated using a TruSeq^®^ DNA PCR-Free sample preparation kit (Illumina, San Diego, CA, USA) according to the manufacturer’s recommendations, and the index codes were added. The quality of the library was then determined by a Qubit@ 2.0 fluorometer (Thermo Fisher Scientific, Carlsbad, CA, USA) and an Agilent Bioanalyzer 2100 system. The library was sequenced on an Illumina NovaSeq platform, and 250-bp paired-end reads were generated. The raw sequence data generated from 16S rRNA MiSeq sequencing were analyzed using Quantitative Insights into Microbial Ecology (QIIME, version 1.17). Gaps in each sequence were discarded from all samples to decrease noise by screening, filtering, and pre-clustering processes. The sequences were clustered into Operational taxonomic units (OTUs) with a cut-off value of 97% similarity by Uparse software (version 7.1, http://drive5.com/uparse/ accessed on 26 April 2020), and chimeric sequences were identified and removed using the UCHIME algorithm. Mothur and SILVA132 (http://www.arb-silva.de/ accessed on 13 December 2017) classifier tools were used to classify all sequences into different taxonomic groups. The functions of the microbial community were predicted using the Functional Annotation of Prokaryotic Taxa (FAPROTAX) method [33].

### 2.8. Statistical Analysis

The GLM procedure of SAS 9.3 (SAS Institute Inc., Cary, NC, USA) was used to analyze the data as a 2 × 2 factorial. DON (0 or 3.8 mg/kg diet), RES (0 or 300 mg/kg diet), and the interactive effect of DON and RES as the fixed effects, with pig identification as the random effects in the model. The pen was regarded as the experimental unit for all analyses. Means and pooled SEM were presented. When the main effect or interaction effect was significant (*p* ≤ 0.05), post hoc testing was conducted using Duncan’s multiple comparison tests, and differences were considered significant when *p* ≤ 0.05.

## 3. Results

### 3.1. Effect of Resveratrol Supplementation on the Redox Status in Piglets Challenged with Deoxynivalenol

As shown in Table 4, compared with non-challenged control piglets, DON-challenged piglets had higher MDA (*p* < 0.01) levels but lower levels of GSH and T-AOC (*p* < 0.01) in the plasma. Compared with diets without RES, RES supplementation significantly increased the levels of GSH and T-AOC (*p* < 0.01) while decreasing MDA (*p* < 0.01) levels in the plasma. Compared with DON exposure alone, Supplemental RES prevented DON-induced increases in MDA levels and reduction in T-AOC in DON-challenged piglets. Additionally, a significant DON × RES interaction was observed for plasma MDA levels (*p* < 0.05).

In ileal mucosa, DON-challenged piglets showed significant reductions in the levels of T-SOD, GSH and T-AOC (*p* < 0.01) and increases in MDA levels (*p* < 0.01) compared with those non-challenged piglets (Table 4). RES supplementation significantly increased the levels of T-SOD and T-AOC (*p* < 0.01) while decreasing MDA levels (*p* < 0.01) compared with diets without RES (Table 4). Compared with DON infection alone, supplementation with RES in DON-challenged piglets effectively alleviated DON-induced increases in MDA levels and increased the levels of T-SOD and T-AOC (*p* < 0.05). In addition, a significant DON×RES interaction was observed for MDA levels (*p* < 0.05) (Table 4).

DON-challenged piglets showed significant decreases in the abundances of superoxide dismutase 1(*SOD1*), glutamate–cysteine ligase catalytic subunit (*GCLC*), glutamate–cysteine ligase modulatory subunit (*GCLM*), heme oxygenase-1 (*HO-1*) and NAD(P)H dehydrogenase, quinone 1 (*NQO-1*) transcripts (*p* < 0.01) in the ileal mucosa compared with those non-challenged piglets (Table 4). Piglets fed diets supplemented with RES showed significant increases in the mRNA expression levels of ileal *SOD1*, *GCLC*, *GCLM*, *HO-1* and *NQO-1* genes (*p* < 0.01) compared with those fed diets without RES (Table 4). Compared with DON challenge alone, supplementation with RES in DON-challenged piglets prevented DON-induced reduction in the abundance of *SOD1* and *GCLC* transcripts (*p* < 0.05). Additionally, a significant DON×RES interaction was observed for the mRNA expression of ileal *SOD1* and *GCLC* genes (*p* < 0.01) (Table 4).

### 3.2. Effect of Resveratrol Supplementation on Pro-Inflammatory Cytokines in Piglets Challenged with Deoxynivalenol

As shown in Table 5, DON exposure significantly increased plasma *IL-1β* (*p* < 0.01) compared with diets without DON. RES supplementation significantly decreased plasma *IL-1β* (*p* < 0.05) compared with diets without RES. Compared with DON challenge alone, supplementation with RES in DON-challenged piglets prevented DON-induced increase in the plasma *IL-1β* protein level (*p* < 0.05).

As for the protein levels of cytokine profiles in the ileum, DON infection induced dramatic increases in the expression of ileal *IL-1β*, *TNF-α* and *IL-6* proteins (*p* < 0.01). Compared with diets without RES, RES supplementation significantly decreased ileal *IL-1β*, *TNF-α* and *IL-6* (*p* < 0.05). Compared with the DON challenge alone, supplementation with RES in DON-challenged piglets reversed DON-induced increases in the levels of *IL-1β*, *IL-6* and *TNF-α* (*p* < 0.05). In addition, a significant DON×RES interaction was observed for the levels of ileal *IL-1β*, *IL-6* and *TNF-α* (*p* < 0.05) (Table 5).

Additionally, DON-challenged piglets showed a higher abundance of ileal *IL-1β, IL-6,* and *TNF-α* transcripts than those of non-challenged piglets (*p* < 0.01). Piglets fed diets supplemented with RES showed significant decreases in the mRNA expression of ileal *IL-1β, IL-6* and *TNF-α* genes(*p* < 0.01) compared with diets without RES (*p* < 0.05). Compared with the DON challenge alone, supplementation with RES in DON-challenged piglets abrogated DON-induced increases in the abundance of *IL-1β*, *IL-6* and *TNF-α* transcripts (*p* < 0.05). In addition, a significant DON×RES interaction for the expression of ileal *IL-1β, IL-6* and *TNF-α* transcripts was observed (*p* < 0.05) (Table 5).

### 3.3. Effect of Resveratrol Supplementation on Expression of Antibacterial Peptide and Mucin in the Ileum of Piglets Challenged with Deoxynivalenol

Compared with those non-challenged piglets, DON significantly decreased mRNA expression of porcine β defensin 1 (*pBD*-1), porcine β defensin 2 (*pBD*-*2*), protegrin 1-5 (*PG1*-*5*) and mucin 2 (*Muc2*) transcripts in DON-treatment ileum (*p* < 0.01). RES supplementation significantly increased the abundance of ileal *pBD*-2 and *Muc2* transcripts compared with diets without RES (*p* < 0.01). Compared with DON challenge alone, supplementation with RES in DON-challenged piglets effectively prevented DON-induced decline in *pBD-2, PG1-5* *and MUC2* gene expression (*p* < 0.05). Additionally, a significant DON×RES interaction for ileal *PG1*-5 gene expression was observed (*p* < 0.01) (Table 6).

At the protein levels, DON-challenged piglets showed significant decreases in ileal *Muc2* and *sIgA* contents (*p* < 0.01) compared with those non-challenged piglets. RES supplementation significantly increased the expression of ileal *Muc2* and *sIgA* proteins compared with diets without RES (*p* < 0.05). Compared with the DON challenge alone, supplementation with RES in DON-challenged piglets effectively reversed the DON-induced reduction in *Muc2* protein expression (*p* < 0.05). In addition, a significant DON × RES interaction for the expression of ileal *Muc2* protein was observed (*p* < 0.05) (Table 6).

### 3.4. Effect of Resveratrol Supplementation on Ileal Morphology in Piglets Challenged with Deoxynivalenol

Histological analysis (Table 7) showed that DON-contaminated diet-induced ileal injury, as reflected by a shortened villus height, decreased villus height to crypt depth ratio (VCR) (*p* < 0.01) compared with the effects of diets lacking DON. RES supplementation significantly increased villus height and VCR (*p* ≤ 0.01) while decreasing crypt depth (*p* < 0.01) in the ileum of weaned piglets compared with diets without RES. Additionally, a significant DON × RES interaction for ileal villus height and VCR was observed (*p* < 0.01).

### 3.5. Effects of Resveratrol Supplementation on the Composition of the Colonic Microbiota of Piglets Challenged with Deoxynivalenol

**Figure 1:**
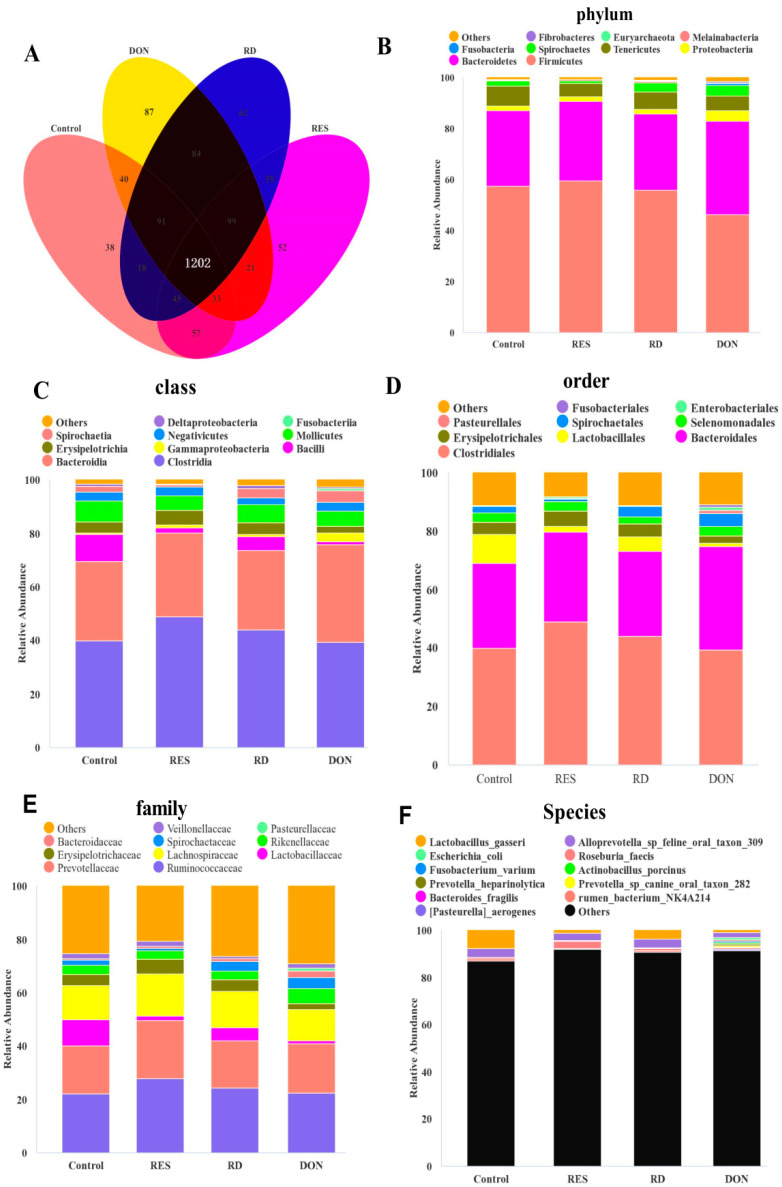
Effect of resveratrol supplementation on the relative abundance of gut microbiota in DON-challenged piglets. (**A**) Venn diagram depicting unique and shared OTU dietary groups. Stacked bar chart representing the relative abundance of colonic bacteria: (**B**) at the phylum level (top 10); (**C**) at the class level (top 10); (**D**) at the order level (top 10); (**E**) at the family level (top 10) and (**F**) at the species level (top 10) in the different treatment groups. DON, deoxynivalenol; DON + RES, combination of deoxynivalenol and resveratrol; RES, resveratrol.

**Figure 2:**
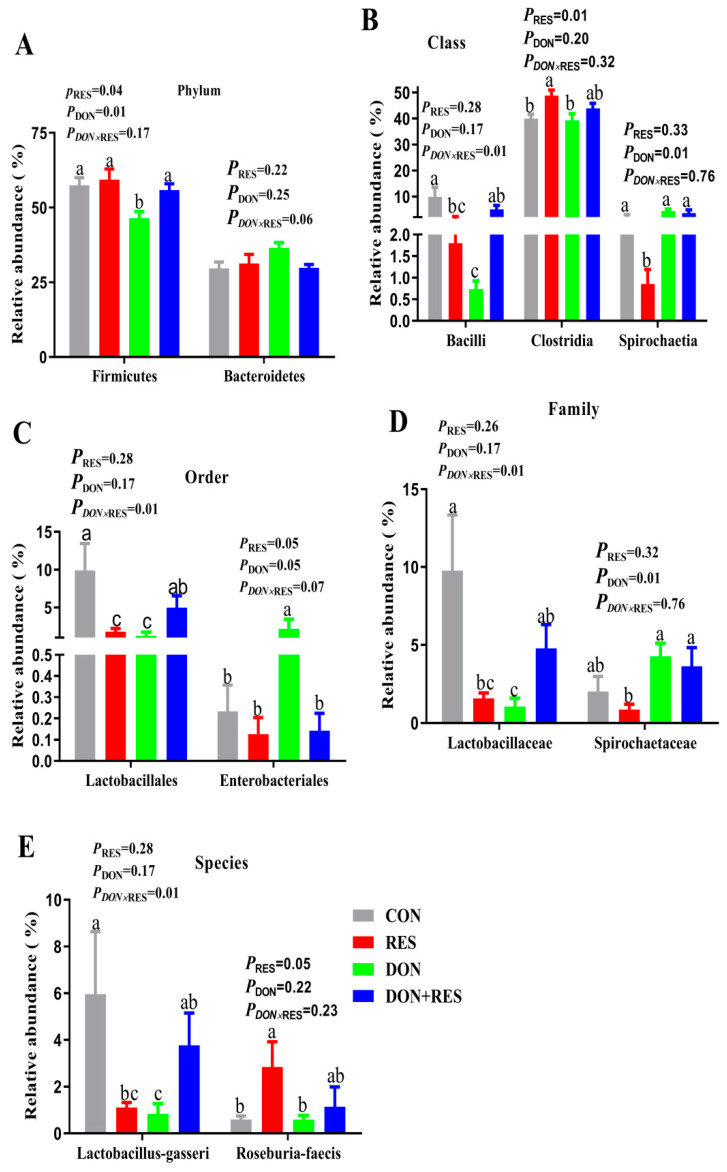
Effect of resveratrol supplementation on the relative abundance of gut microbiota in DON-challenged piglets. The significantly different microbial (**A**) at phylum level (top 10), (**B**) at class level (top 10), (**C**) at order level (top 10), (**D**) at family level (top 10) and (**E**) at species level (top 10) were showed in the different treatment groups. Data are presented as means and pooled SEM, n  =  8; labeled means in a row without a common letter differ, *p * ≤  0.05; CON, control; DON, deoxynivalenol; DON + RES, combination of deoxynivalenol and resveratrol; RES, resveratrol.

As shown in the Venn diagram analysis of the OTUs, 38, 87, 62 and 52 unique OTUs were identified in the Control, DON, RES, and DON + RES groups, respectively, and 1202 OTUs were shared among the four treatment groups (Figure 1A). DON-challenged piglets showed an increase in bacterial richness and diversity indices for alpha-diversity compared with those non-challenged piglets, as reflected by the significant increase in the observed species, Chao1 and ACE, Shannon indexes (*p* < 0.05) (Table 8). However, no significant DON×RES interaction or main effect of RES on the alpha-diversity indices was observed. As shown in Figure 1 and Figure 2, DON exposure significantly increased the relative abundances of class *Spirochaetia*, order *Enterobacteriales* and family *Spirochaetaceae* while decreasing the abundance of phylum *Firmicutes* (*p* ≤ 0.05). The relative abundance of class *Clostridia* and species *Roseburia faecis* was significantly elevated, but the relative abundance of order *Enterobacteriales* was significantly reduced by the main effect of RES (*p* ≤ 0.05). Compared with the DON challenge alone, the relative abundance of phylum *Firmicutes*, class *Bacilli*, order *Lactobacillales*, family *Lactobacillaceae* and species *Lactobacillus gasseri* was increased, while decreasing order *Enterobacteriales* (*p* ≤ 0.05) in DON-challenged piglets fed a RES-supplemented diet compared with those in DON-challenged alone piglets. In addition, a significant DON × RES interaction was observed for the relative abundances of class *Bacilli*, order *Lactobacillales*, family *Lactobacillaceae* and species *Lactobacillus gasseri* (*p* < 0.05).

### 3.6. Microbiota Functional Prediction Analysis

**Figure 3:**
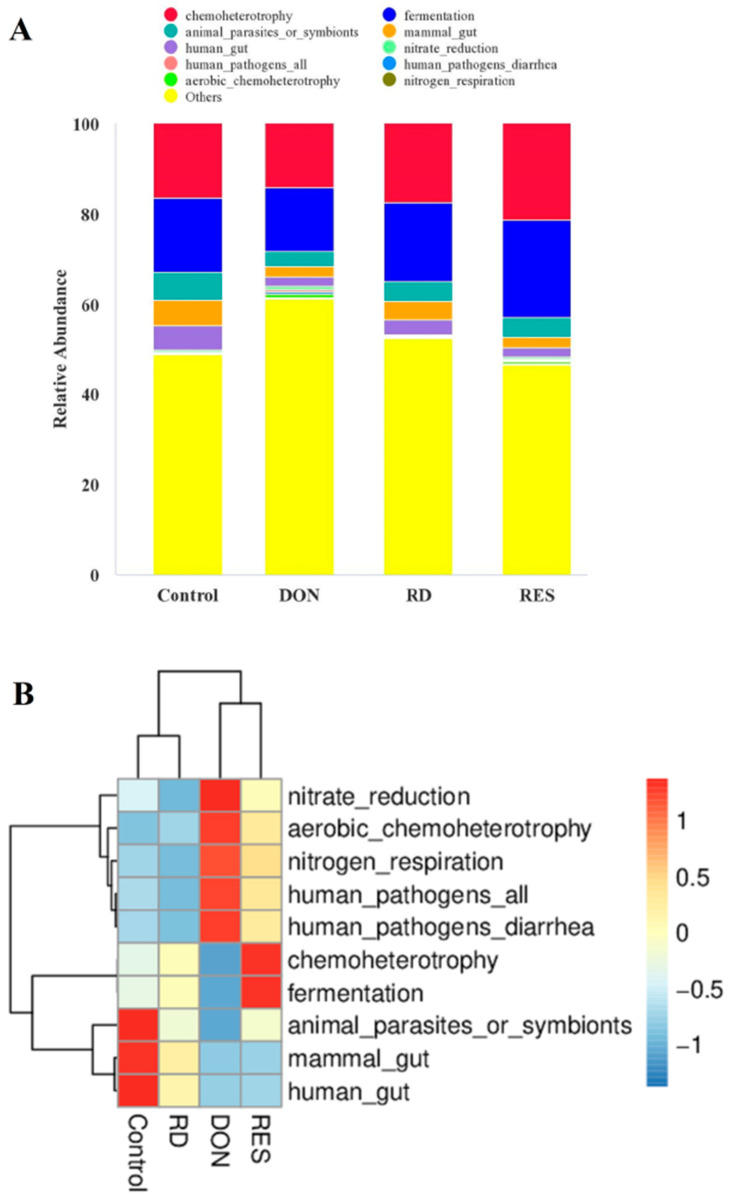
Histogram (**A**) and Heatmap (**B**) showing the predicted function potentials of intestinal microbiota in the piglets with different treatments through FAPROTAX prediction.

FAPROTAX was applied to conduct the prediction of metagenome functional contents (Figure 3). The most dominant functions of the intestinal microbiota among the four treatment groups were mainly chemoheterotrophy, fermentation, animal parasites or symbionts, mammal gut and human gut, with a total relative abundance of approximately 50%. Compared to diets without RES, RES supplementation increased bacterial abundance for chemoheterotrophy and fermentation in the heatmap. The heatmap result also revealed that the gut microbes differentially expressed in the DON-challenged piglets were mostly represented by nitrate reduction, aerobic chemoteterotrophy, nitrogen respiration, human pathogens—all, and human pathogens—diarrhea. Additionally, RES supplementation in DON-challenged piglets prevented DON-induced increase in nitrate reduction, aerobic chemoteterotrophy, nitrogen respiration, human pathogens—all, and human pathogens—diarrhea.

### 3.7. Correlation Analysis of the Gut Microbiota and Variables Related to Intestinal Barrier Function, Inflammation and Oxidative Damage

**Figure 4:**
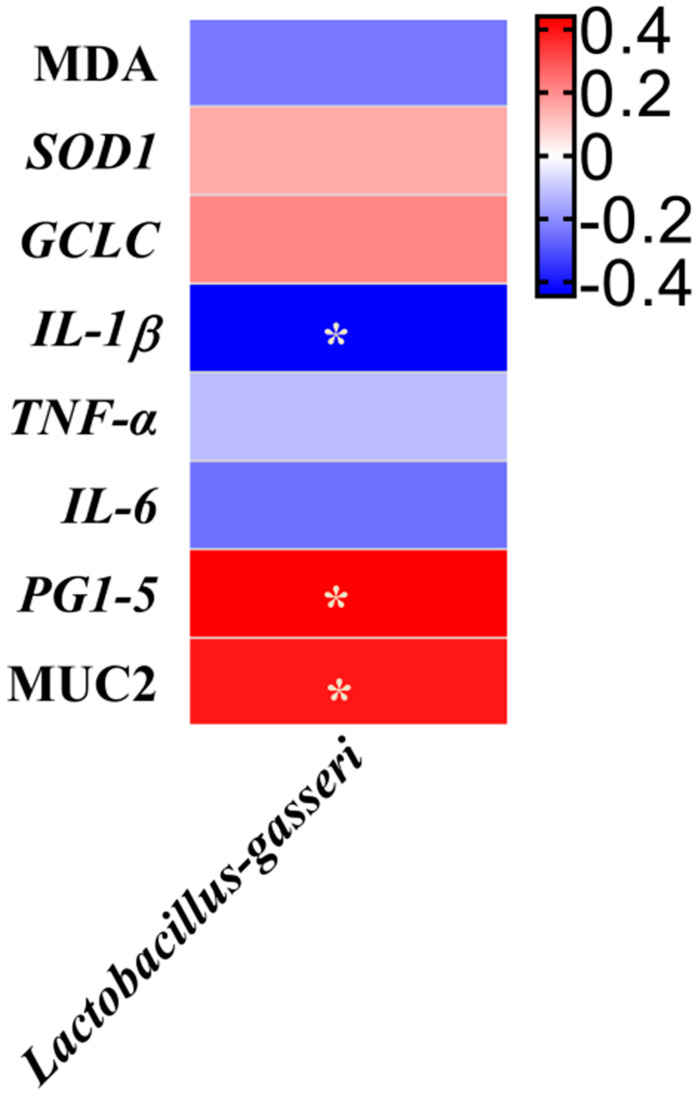
Heatmap of Pearson correlation coefficients between the intestinal inflammation and oxidative damage and the relative abundance of Lactobacillus gasseri affected by RES and DON. * Significant correlation: * *p* ≤ 0.05. Red shading with *p* ≤ 0.05 represents a significant positive correlation; blue shading with *p* ≤ 0.05 represents a significant negative correlation; white represents no correlation. *GCLC*, glutamate–cysteine ligase catalytic subunit; *IL-1β*, interleukin 1 beta; *IL-6*, interleukin 6; MDA, malondialdehyde; *Muc2*, mucin 2; PG1-5, protegrin 1-5; *TNF-α*, tumor necrosis factor-alpha.

A Pearson correlation analysis was used to determine the correlations between variables related to intestinal inflammation and oxidative damage and the relative abundance of *Lactobacillus gasseri* (Figure 4). The relative abundance of *Lactobacillus gasseri* showed positive correlations with the *PG1-5* mRNA expression and the protein expression of *MUC2* but was negatively associated with the *IL-1β* mRNA expression in the ileum (*p* ≤ 0.05).

## 4. Discussions

Previous studies demonstrated that DON induced severe oxidative stress in IPEC-J2 cells, as reflected by increased ROS levels and dramatic decreases in the abundance of *SOD1, GCLC* and *GCLM* transcripts [8,10]. Similarly, an in vivo study showed that DON elevated MDA levels in the blood and small intestine, which is a sensitive indicator of oxidative stress [18]. Consistently, the results in the present study observed that DON-challenged piglets exhibited elevated MDA levels and decreased GSH and T-AOC levels in the plasma, and increased MDA levels and decreased T-SOD, GSH and T-AOC levels in the ileum. Additionally, DON exposure also significantly induced the decline in the abundance of ileal *GCLC*, *GCLM*, *HO-1*, *NQO1* and *SOD1* transcripts in the present study. Previous studies showed that polyphenols perform effective antioxidative effects in intestinal epithelial cell culture and weaned piglet studies [12,13,14,34,35]. Consistent with these observations, the results in this study showed that RES supplementation significantly suppressed plasma and ileal MDA levels while increasing the levels of GSH and T-AOC in the plasma and the contents of T-SOD and T-AOC in the ileum. Moreover, providing DON-challenged piglets with diets supplemented with RES effectively reduced MDA levels in the plasma and ileum. Similarly, a study by Cao et al. suggested that dietary RES alleviated oxidative stress in diquat-challenged piglets, as indicated by decreased MDA levels in the jejunum [18]. As expected, the results in the present study revealed that RES supplementation also upregulated the expression of antioxidant genes, including *GCLC* and *SOD1,* in the ileum of DON-challenged piglets [10,36,37]. Consistent with the present observation, a previous study showed that the abundance of *SOD1, GCLC* and *GCLM* transcripts in IPEC-J2 cells were suppressed by DON, and these effects were blocked when cells were pretreated with RES [10]. Collectively, the results in the present study suggested that RES effectively attenuated oxidative stress in DON-challenged piglets.

Numerous studies found that DON challenge also induces intestinal inflammation, which impairs intestinal health [8,10,29,38]. Meanwhile, it was convincingly demonstrated that RES effectively suppress experimentally induced inflammation in the model of swine and porcine intestinal epithelial cell [10,11,15,16,39]. A previous study reported that supplementation with RES prevented *TNF-α* production in piglets infected with rotavirus [40]. Consistent with these findings, our results in the present study showed that supplemental RES in DON-challenged piglets reversed the increases in the mRNA expression of ileal *IL-1β, IL-6 and TNF-α* genes and their proteins induced by DON exposure, suggesting that supplementation with RES in DON-challenged piglets can effectively abrogate the increase in intestinal inflammation caused by DON.

Antimicrobial proteins, which are mainly secreted by intestinal epithelial cells, play an important role in protecting against pathogen invasion into the underlying epithelium [41,42]. In this study, the DON challenge decreased the expression of *pBD1, pBD2* and *PG1-5* genes in the ileum of weaned pigs. Consistently, a previous study found that a DON-contaminated diet induced a significant decline in the abundance of intestinal *pBD1, pBD2* and *pBD3* transcripts and the protein expression of pBD3, resulting in the impairment of intestinal immune function and growth performance of weaned piglets [38]. However, pretreatment with RES effectively reversed the reduction in *hBD-2* gene expression and the increase in *IL-8* mRNA expression induced by Streptococcus in A549 cells in a SIRT1-dependent manner [43]. Similarly, in the present study, the results showed that supplementation with RES in DON-challenged reversed the decline in the expression of the *pG1-5* gene, suggesting that RES played an important role in intestinal defense response. To our best knowledge, this study is the first to investigate the effects of dietary RES supplementation on antimicrobial proteins in DON-challenged weaned piglets.

A previous study demonstrated that knockout mice lacking the expression of *Muc2* protein, which is produced by epithelial goblet cells and is the major component of intestinal mucin, developed spontaneous colitis, indicating that *Muc2* played an important role in the protection of the gut barrier [44]. It was reported that the DON-contaminated diet suppressed the expression of the duodenal *Muc2* gene and its protein in broiler chickens, which induced the alteration of the mucin monosaccharide composition [45]. Consistent with this finding, we observed that DON exposure significantly decreased the expression of ileal *Muc2* protein. However, supplementation with RES in DON-treated piglets effectively reversed the decline in *Muc2* protein expression, suggesting that RES promoted the synthesis of *Muc2* protein and thus enhanced the mucosal layer, contributing to protecting the intestinal epithelium from injury. To the best of our knowledge, we are the first to investigate the effects of dietary RES supplementation on mucin protein expression in DON-challenged weaned piglets.

Villus height, crypt depth, and VCR are regarded as useful indices to evaluate intestinal health and functions [46]. In the present study, DON exposure decreased villus height and VCR in the ileum, indicating that DON induced severe intestinal mucosal damage. Consistently, Wang et al. found that a DON-contaminated diet reduced villus height in the duodenum and jejunum and decreased VCR in all regions of the intestine of piglets [33]. Several previous studies indicated that polyphenols increased villus height and VCR in pigs [34,35]. Additionally, a study by Zhang et al. reported that supplemental RES attenuated radiation-induced intestinal injury [47]. Consistent with these observations, we found that supplementation with RES in DON-challenged reversed the decreases in villus height and VCR of the ileum induced by DON exposure in this study, suggesting that dietary RES supplementation alleviated intestinal morphology damage induced by DON.

Gut microbiota plays an important role in maintaining intestinal barrier function homeostasis. In the present study, RES supplementation increased the abundance of class *Bacilli*, order *Lactobacillales*, family *Lactobacillaceae* and species *Lactobacillus gasseri* in DON-challenged piglets. The species *Lactobacillus gasseri* belongs to the phylum *Firmicutes*, class *Bacilli*, order *Lactobacillales*, family *Lactobacillaceae* and genus *Lactobacillus*. A previous study demonstrated that *Lactobacillus gasseri* protects the intestine from dextran sodium sulfate (DSS)-induced colitis and maintains immune homeostasis by increasing the antioxidant response [48], which was supported by the increased expression of GHS, T-SOD and T-AOC. These findings suggested that RES supplementation in DON-challenged piglets increased the abundance of *Lactobacillus gasseri*, which may contribute to alleviating intestinal barrier injury.

## 5. Conclusions

The results in the present study indicated that RES supplementation in DON-challenged piglets efficiently attenuated ileal inflammation and oxidative damage by decreasing the gene and protein expression of *IL-1β*, *IL-6*, *TNF-α* and MDA levels and upregulating the expression of *SOD1, GCLC, pG1-5* genes and *Muc2* protein in the ileum, and increased the abundance of *Lactobacillus gasseri* in the colon, and thereby alleviated DON-induced intestinal barrier injury.

## Figures and Tables

**Table 1:** The levels of main mycotoxins in feed.

Item, μg/kg	Basal Feed	Contaminated Feed	Limit of Detection
DON	247	3790	100
AFB	undetected	undetected	2
ZEN	94	102	10
OTA	16	21	10

**Table 2:** Ingredient composition and nutrient levels of the basal diet (%, as-fed basis).

Item	
Ingredient composition, %	
Corn	34.00
Expanded corn	14.72
Soybean meal	10.0
Expanded soybean	8.50
Fishmeal	4.00
Low protein whey powder	11.00
Soybean hull	5.00
Plasma protein powder	4.00
Soybean oil	1.35
Sucrose	2.00
CaHPO4	1.20
Limestone powder	0.65
NaCL	0.45
Choline chloride 50%	0.20
L-Lysine HCl	0.82
DL-Methionine	0.25
L-Threonine	0.30
Trp	0.06
Premix ^1^	1.50
Total	100
Nutrient levels ^2^	
DE, kcal/kg	3516.62
CP%	19.26
SID Lys%	1.55
SID Met + Cys%	0.78
SID Thr%	0.88
SID Trp%	0.25

^1^ Supplied per kilogram of complete diet:: acidifier 4 g, artificial sweetener 0.2 g, feed flavor 1 g, phytase 0.2 g, compound enzyme preparation 4 g, ZnO 2 g, zeolite powder 1 g, vitamin A 12400 IU, vitamin D3 2800 IU, vitamin E 130 mg, vitamin K 5 mg, vitamin B1 3 mg, vitamin B2 10 mg, vitamin B3 40 mg, vitamin B5 15 mg, vitamin B6 8 mg, vitamin B12 40 μg, folic acid 1 mg, biotin 0.08 mg, Fe (FeSO4·H2O) 120 mg, Cu (CuSO4·5H2O) 16 mg, Mn (MnSO4·H2O) 70 mg, Zn (ZnSO4·H2O) 120 mg, I (CaI2O6) 0.7 mg, Se (Na2SeO3) 0.48 mg. ^2^ Nutrient levels are calculated based on the NRC (2012) database and Tables of feed composition and nutritive values in China (the values of sucrose are from Tables of feed composition and nutritive values in China, and others are from NRC (2012)). CP, crude protein; DE, digestible energy; SID, standardized ileal digestibility.

**Table 3:** Primer sequences used in this study.

Genes	Sequences, 5′–3′		Product Size, Bp	GenBank Accession
*β-actin*	Forward Reverse	CATCGTCCACCGCAAAT TGTCACCTTCACCGTTCC	210	NC_010445
*SOD1*	Forward Reverse	GAGACCTGGGCAATGTGACT CTGCCCAAGTCATCTGGTTT	139	NM_001190422.1
*GCLC*	Forward Reverse	CAAACCATCCTACCCTTTGG ATTGTGCAGAGAGCCTGGTT	172	XM_003482164.4
*GCLM*	Forward Reverse	GATGCCGCCCGATTTAACTG ACAATGACCGAGTACCGCAG	177	XM_001926378.4
*HO-1*	Forward Reverse	CGCTCCCGAATGAACACTCT GCGAGGGTCTCTGGTCCTTA	148	NM_001004027.1
*NQO-1*	Forward Reverse	ATCACAGGTAAACTGAAGGACCC TGGCAGCGTATGTGTAAGCA	229	NM_001159613.1
*IL-1β*	Forward Reverse	CTCCAGCCAGTCTTCATTGTTC TGCCTGATGCTCTTGTTCCA	230	NM_214055.1
*IL-6*	Forward Reverse	TACATCCTCGGCAAAATC TCTCATCAAGCAGGTCTCC	168	NM_001252429.1
*Muc2*	Forward Reverse	CTGCTCCGGGTCCTGTGGGA CCCGCTGGCTGGTGCGATAC	100	XM_007465997.1
*pBD1*	Forward Reverse	ACCGCCTCCTCCTTGTATTC CACAGGTGCCGATCTGTTTC	150	NM_213838.1
*pBD2*	Forward Reverse	CCAGAGGTCCGACCACTACA GGTCCCTTCAATCCTGTTGAA	88	AY506573.1
*PG1-5*	Forward Reverse	GTAGGTTCTGCGTCTGTGTCG CAAATCCTTCACCGTCTACCA	166	XM_005669497.2

*β-actin*, actin beta; *IL-1β*, interleukin 1 beta; *IL-6,* interleukin 6; *GCLC*, glutamate–cysteine ligase catalytic subunit; *GCLM*, glutamate–cysteine ligase modulatory subunit; *GSH*, glutathione; *HO-1*, heme oxygenase-1; *Muc2*, mucin 2; *NQO-1*, NAD(P)H dehydrogenase, quinone 1; *pBD1*, porcine β defensin 1; *pBD2*, porcine β defensin 2; *PG1*-*5*, protegrin 1–5; *SOD1*, superoxide dismutase 1; *TTNF-α*, tumor necrosis factor-alpha.

**Table 4:** Effect of resveratrol supplementation on the redox status in the plasma and ileum of DON-challenged piglets ^1^.

Variable	Treatments				Pooled SEM	*p* Value		
	CON	DON	RES	DON + RES		DON	RES	RES × DON
The levels of antioxidant/oxidant indices in the plasma								
MDA (nmol/mL)	1.96 c	3.31 a	1.80 c	2.58 b	0.12	<0.01	<0.01	0.02
T-SOD (U/mL)	60.57	53.67	62.39	61.84	2.91	0.21	0.10	0.28
GSH (mg/L)	4.24 b	2.80 c	5.59 a	3.38 c	0.24	<0.01	<0.01	0.17
T-AOC (U/mL)	2.31 b	1.56 c	3.17 a	2.01 b	0.15	<0.01	<0.01	0.20
The contents of antioxidant/oxidant indices in the ileum, μmol/g protein								
MDA	0.52 c	1.00 a	0.43 c	0.68 b	0.04	<0.01	<0.01	0.01
T-SOD	58.24 b	39.57 d	77.00 a	50.77 c	2.17	<0.01	<0.01	0.09
GSH	5.13 ab	4.21 c	5.68 a	4.38 bc	0.26	<0.01	0.17	0.48
T-AOC	0.49 ab	0.31 c	0.56 a	0.44 b	0.03	<0.01	<0.01	0.29
The expression of antioxidative genes in the ileum								
*SOD1*	1.01 b	0.56 c	2.31 a	1.03 b	0.10	<0.01	<0.01	<0.01
*GCLC*	1.01 b	0.72 b	1.94 a	0.82 b	0.14	<0.01	0.01	<0.01
*GCLM*	1.01 b	0.41 c	1.73 a	0.78 b	0.13	<0.01	<0.01	0.17
*HO-1*	1.01 b	0.70 c	1.40 a	0.78 c	0.08	<0.01	<0.01	0.07
*NQO-1*	1.01b	0.69 c	1.54 a	0.89 bc	0.10	<0.01	0.01	0.11

^1^ Values are presented as means and pooled SEM, n = 8/treatment; labeled means in a row without a common letter differ, *p* ≤ 0.05; CON, control; DON, deoxynivalenol; DON + RES, combination of deoxynivalenol and resveratrol; *GCLC*, glutamate–cysteine ligase catalytic subunit; *GCLM*, glutamate–cysteine ligase modulatory subunit; *HO-1*, heme oxygenase-1; MDA, malondialdehyde; RES, resveratrol; *NQO-1*, NAD(P)H dehydrogenase, quinone 1; SOD, superoxide dismutase; SEM, standard error of the mean; T-AOC, total antioxidant capacity.

**Table 5:** Effects of resveratrol on the expression of cytokine in the plasma and ileal mucosa of weaned piglets challenged with deoxynivalenol ^1^.

Variable	Treatments				Pooled SEM	*p* Value		
	CON	DON	RES	DON + RES		DON	RES	RES × DON
The protein levels of pro-inflammation cytokines levels in the plasma, ng/L								
*IL-1β*	194.74c	227.01a	189.00c	211.92b	4.38	<0.01	0.03	0.30
*IL-6*	398.28	406.59	394.42	410.25	14.80	0.42	0.10	0.80
*TNF-α*	61.37	63.83	59.06	60.09	2.11	0.41	0.16	0.73
The pro-inflammation cytokines contents in the ileum, ng/g protein								
*IL-1β*	20.40c	38.41a	17.45c	32.13b	2.21	<0.01	0.05	0.03
*IL-6*	25.44c	38.09a	21.45c	32.80b	2.56	<0.01	0.03	0.04
*TNF-α*	14.12b	19.44a	9.75c	15.45b	1.25	<0.01	<0.01	0.03
The gene expression of pro-inflammation in the ileum								
*IL-1β*	1.01bc	1.69a	0.86c	1.22b	0.07	<0.01	<0.01	0.04
*IL6*	1.01c	2.01a	0.66d	1.25b	0.08	<0.01	<0.01	0.01
*TNF-α*	1.02b	1.66a	0.78c	1.08b	0.07	<0.01	<0.01	0.02

^1^ Values are presented as means and pooled SEM, n = 8/treatment; labeled means in a row without a common letter differ, *p* ≤ 0.05; CON, control; DON, deoxynivalenol; DON + RES, combination of deoxynivalenol and resveratrol; *IL-1β*, interleukin 1 beta; *IL-6*, interleukin 6; RES, resveratrol; SEM, standard error of the mean; *TNF-α*, tumor necrosis factor-alpha.

**Table 6:** Effects of resveratrol on the expression of genes related to innate immunity in the ileal mucosa of weaned piglets challenged with deoxynivalenol ^1^.

Variable	Treatments				Pooled SEM	*p* Value		
	CON	DON	RES	DON + RES		DON	RES	RES × DON
Gene expression								
*pBD-1*	1.02 a	0.44 b	1.05 a	0.57 b	0.06	<0.01	0.16	0.42
*pBD-2*	1.02 a	0.41 c	1.08 a	0.71 b	0.06	<0.01	<0.01	0.07
*pG1-5*	1.01 a	0.54 c	0.96 a	0.77 b	0.05	<0.01	0.09	<0.01
*Muc2*	1.01 ab	0.71 c	1.07 a	0.92 b	0.05	<0.01	<0.01	0.18
Protein expressions								
*Muc2*	9.23 a	6.89 b	10.09 a	8.54 a	0.51	<0.01	0.02	0.04
*sIgA*	491.59 a	354.63 c	517.31 a	421.63 b	20.47	<0.01	0.03	0.32

^1^ Values are presented as means and pooled SEM, n = 8/treatment; labeled means in a row without a common letter differ, *p* < 0.05; CON, control; DON, deoxynivalenol; DON + RES, combination of deoxynivalenol and resveratrol; *Muc2*, mucin 2; *pBD1*, porcine β defensin 1; *pBD2*, porcine β defensin 2; *PG1-5*, protegrin 1–5; RES, resveratrol; SEM, standard error of the mean; *sIgA*, secretory immunoglobulin A.

**Table 7:** Effect of resveratrol supplementation on intestinal morphology and tight junction proteins in DON-challenged piglets ^1^.

Variable	Treatments				Pooled SEM	*p* Value		
	CON	DON	RES	DON + RES		DON	RES	DON × RES
Villus height, μm	382 b	354 b	438 a	359 b	11	<0.01	0.01	0.03
crypt depth, μm	292 a	278 a	238 b	266 ab	12	0.54	0.01	0.09
VCR	1.33 b	1.29 b	1.85 a	1.36 b	0.08	<0.01	<0.01	<0.01

^1^ Values are presented as means and pooled SEM, n = 8/treatment; labeled means in a row without a common letter differ, *p* < 0.05; CON, control; DON, deoxynivalenol; DON + RES, combination of deoxynivalenol and resveratrol; SEM, standard error of the mean; VCR, villus height to crypt depth ratio.

**Table 8:** Effect of resveratrol supplementation on the alpha-diversity of gut microbiota in DON-challenged piglets ^1^.

Variable	Treatments				Pooled SEM	*P* Value		
	CON	DON	RES	DON + RES		DON	RES	DON × RES
Alpha-diversity index								
ACE	916 b	1028 a	890 b	969 ab	88	<0.01	0.17	0.60
Shannon	7.25 b	7.67 a	7.01 b	7.37 ab	0.38	0.01	0.17	0.53
species	883 b	970 a	829 b	914 ab	82	<0.01	0.17	0.66
Chao1	1005 ab	1023 a	886 b	964 b	84	0.01	0.17	0.53

^1^ Values are presented as means and pooled SEM, n = 8 per treatment; labeled means in a row without a common letter differ, *p* ≤ 0.01. ACE, abundance coverage-based estimator; CON, control; DON, deoxynivalenol; DON + RES, combination of deoxynivalenol and resveratrol; SEM, standard error of the mean.

## Data Availability

Not applicable.
